# Geographic Distribution and Niche Divergence of Two Stinkbugs, *Parastrachia japonensis* and *Parastrachia nagaensis*

**DOI:** 10.1673/031.013.10201

**Published:** 2013-10-05

**Authors:** Gengping Zhu, Guoqing Liu, Wenjun Bu, Jerzy A. Lis

**Affiliations:** 1 Tianjin Key Laboratory of Animal and Plant Resistance, College of Life Sciences, Tianjin Normal University, Tianjin 300387, China; 2 Institute of Entomology, College of Life Sciences, Nankai University, Tianjin 300071, China; 3 Department of Biosystematics, Opole University, Oleska 22, 45–052 Opole, Poland

**Keywords:** ecological niche, ecological niche modeling, potential distribution, principle component analysis

## Abstract

Parastrachiidae is a small stinkbug family containing only one genus and two species, *Parastrachia* *japonensis* (Scott) (Hemiptera: Heteroptera: Pentatomoidea) and *Parastrachia nagaensis* Distant. The geographic distribution of the genus has been poorly studied. Niche conservatism refers to that idea that closely related species are more ecologically similar than would be expected, whereas niche divergence predicts they occupy distinct niche spaces. The existence of only two species within one genus suggests niche conservatism or differentiation might exist among them. Herein, the distribution of the genus was mapped, potential distributions were predicted using ecological niche modeling, and climate spaces occupied by the two species were identified and compared. Our outlined map supports the general spreading route proposed by Schaefer et al. The potential distributions suggest that the genus’ range could extend beyond its presently known distribution, and further investigation into this area could aid in their conservation, particularly *P. nagaensis*. The niche space inferred by ecological niche modeling suggests the two species do not occupy identical habitat, but the differences between their models could simply be due to the differential availability of habitat in the different regions that they occupy.

## Introduction

It is widely accepted that ecology plays an important role in speciation (Rice et al. 2003; Rissler and Apodaca 2007; Pyron and Burbrink 2009). Generally, the speciation event takes place in geographic dimensions and may not be accompanied by ecological innovation (Peterson et al. 1999; Pyron and Burbrink 2009; McCormack et al. 2010; Peterson 2011). When populations enter into a new environment, however, the ecological niche of a species might diverge to adapt to the novel environment, and subsequent natural selection might promote this process and facilitate speciation (Graham et al. 2004; Wiens and Graham 2005; Sánchez-Fernández et al. 2011). Ecological niche differences among different species can be visualized and analyzed to assess the likely ecological and evolutionary forces that shape the species’ geographical distributions and habitat preferences (Graham et al. 2004; Wiens and Graham 2005; Raxworthy et al. 2007; Sánchez-Fernández et al. 2011).

Parastrachiidae is a small stinkbug family containing only one genus and two species, *Parastrachia japonensis* (Scott) (Hemiptera: Heteroptera: Pentatomoidea) and *Parastrachia* *nagaensis* Distant. The genus has attracted the attention of some heteropterists due to its unstable position in Pentatomoidea (i.e., in different families or subfamilies) (Goel and Schaefer 1970; Schaefer et al. 1988; Gapud 1991; Hasan and Kitching 1993; Hasan and Nasreen 1994; Schuh and Slater 1995), and the maternal care behavior of *P*. *japonensis* (Tsukamoto et al. 1994; Filippi et al. 1995a, b, 2000a, b, 2001; Nomakuchi et al. 1998, 2001; Hironaka et al. 2003a, b, 2005, 2007). The genus was raised to the family level (Sweet and Schaefer 2002), which was widely accepted (Grazia et al. 2008; Pluot-Sigwalt and Lis 2008; Lis and Ziaja 2010). However, the geographic distribution of the genus throughout the world, and especially in China, has been poorly studied (Schaefer et al. 1988; Schaefer and Kikuhara 2007). The fact that there are only two species within one genus suggests niche conservatism or differentiation might exist among them.

Principle component analysis (PCA) and ecological niche modeling (ENM) are two approaches that have been widely used to study niche conservatism and differentiation (Broennimann et al. 2007; Fitzpatrick et al. 2007; Warren et al. 2008; Rödder and Lötters 2009; McCormack et al. 2010; Medley 2010; Zhu et al. 2012). PCA uses pooled environmental variables associated with species occurrence to reveal reduced significant components that account for the observed distribution. The defining niche space of reduced dimension allows investigation of niche conservatism and differentiation. ENM seeks to characterize environmental conditions that are suitable for the species, and then to identify where suitable environments are distributed spatially (Pearson 2007). ENM has been widely used in biological responses to climate change, setting conservation priorities, and the study of evolutionary biology (Guisan and Thuiller 2005; Raxworthy et al. 2007; Rissler and Apodaca 2007; Martínez-Gordillo et al. 2010).

In this study, the global distribution of the genus was mapped with many records, especially in China, potential distributions were predicted using ENM, and niche spaces occupied by the two species were identified and compared using PCA and ENM. The ecological niche of a species here can be defined as “a set of environmental conditions under which it is able to maintain populations without immigrational subsidy” (Grinnell 1917, 1924). This study also highlighted the correlative approach for biodiversity conservation, especially for the localized endemic species.

**Table 1.**
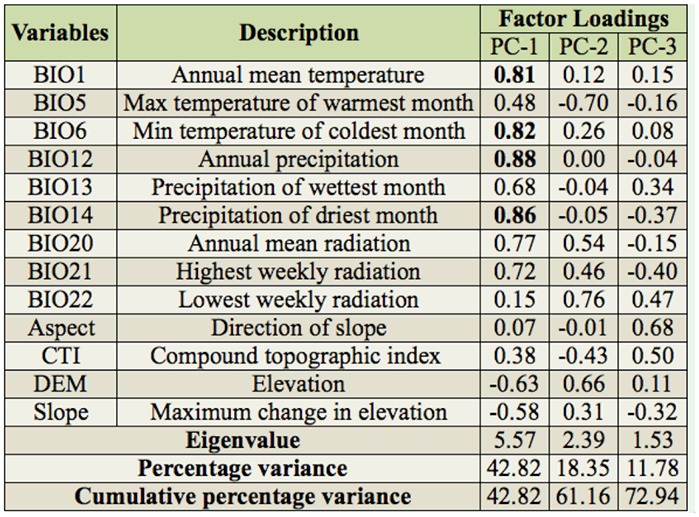
Principal components analysis of 13 environmental variables associated with occurrences of the genus *Parastrachia*, eigenvalues for significant variables (> 0.8) are in bold.

## Materials and Methods

### Occurrence data

**Supplementary Figure 1.**
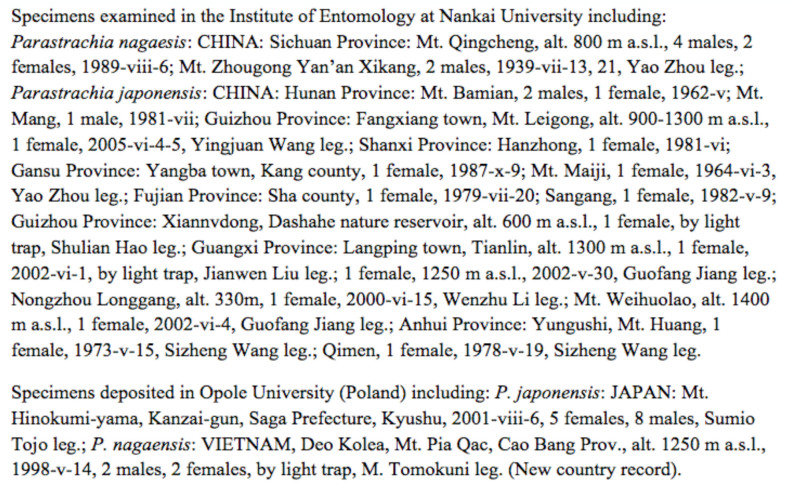
Specimens examined in the Institute of Entomology at Nankai University

Occurrence records were assembled from existing literature (Tsukamoto and Tojo 1992; Hironaka et al. 2003a; Schaefer and Kikuhara 2007; Hosokawa et al. 2010) and our specimens (see Supplementary Figure 1). Localities were georeferenced in Google Maps (http://maps.google.com). Gazetteer of China (1997), or BioGeomancer (http://bg.berkeley.edu/latest/), then mapped in ArcGIS 10 (ESRI 2006). A total of 34 and 6 occurrence records were prepared for *P. japonensis* and *P. nagaensis* respectively.

### Environmental variables

Environmental variables were selected by considering climate and topography factors that might affect the genus' distribution (Tsukamoto et al. 1994; Filippi et al. 1995a, b, 2000a, b, 2001; Nomakuchi et al. 1998, 2001; Hironaka et al. 2003a, b, 2005, 2007) (Table 1). Annual trends and extreme or limiting bioclimatic variables were chosen. Among the variables, 6 (i.e., BIO 1, 5, 6, 12, 13, 14) were summarizing aspects of temperature and precipitation and were obtained from WorldClim (Hijmans et al. 2005), and 3 (i.e., BIO 20, 21, 22) were summarizing aspects of radiation from CliMond (Kriticos et al. 2011). Topography variables represented by elevation, slope, aspect, and compound topographic index were derived from the US Geological Survey's HYDRO1k Elevation Derivative Database (USGS 2001). Variables at a resolution of 2.5 min were used for model calibration.

### Principle component analysis

PCA was used to explore significant variables that related to the genus'distribution and to compare niche spaces occupied by the 2 species. After superimposing the occurrence data on bioclimate and topography grids, values for each record were extracted in ArcGIS 10. A correlation matrix of 40 × 13 was prepared for the PCA performed in SPSS 19 (IBM 2009). To facilitate data visualization, the occurrence records were pooled for *P. nagaensis* and *P*. *japonensis* respectively.

### Ecological niche modeling

A wide range of methods have been explored for ENM. Among them, the maximum entropy algorithm implemented in the Maxent software (Phillips et al. 2004, 2006; Elith et al. 2011) generally performs better than other algorithms (Elith et al. 2006; Phillips et al. 2006; Ortega-Huerta and Peterson 2008). Maximum entropy is a machine-learning technique that predicts species distributions by using detailed environmental variables associated with species occurrence. It follows the principle of maximum entropy and spreads out probability as uniformly as possible, but subject to the caveat that they must match empirical information such as known presence (Phillips et al. 2004, 2006). Maxent is less sensitive to sample size (Wisz et al. 2007) and can be applied to sample sizes as small as five (Pearson et al. 2007). The default convergence threshold (10^-5^), maximum number of iterations (500), and the logistic output with suitability values ranging from 0 (unsuitable habitat) to 1 (optimal habitat) were adopted. A jack-knife procedure was used to evaluate the relative importance of each predictor variable and the ability to correctly predict new occurrences in the model (Pearson et al. 2007).

To visualize niche in ecological dimensions, environmental grids (including annual mean temperature and annual precipitation) and the final predictions were extracted from a mask built using minimum convex polygon for the genus in Hawth's Tools (Beyer 2004) and then combined for each species in ArcGIS 10. The associating attribute tables were imported into SPSS 19, where data density was reduced by selecting a random 10% of the records. These reduced tables were then exported to Microsoft Excel, where scatter plots were prepared for visualization (e.g., Kambhampati and Peterson 2007; Donalisio and Peterson 2011).

### Model evaluation

For *P. japonensis*, the area under the curve (AUC) of the receiver operating characteristic plot and omission rate were adopted for model evaluation. AUC values range from 0 to 1, where 1 is a perfect fit. Useful models produce AUC values of 0.7–0.9, and models with “good discriminating ability” produce AUC values above 0.9 (Swets 1988). The AUC of the receiver operating characteristic plot is a threshold-independent measure of model accuracy, which juxtaposes correct and incorrect predictions over a range of thresholds. Since AUC is sensitive to background size for sampling pseudoabsence data, the background was set as the minimum square area that covered all the occurrences of *P. japonensis*. The omission rate at the 10th percentile training presence threshold was also adopted for model evaluation; the 10th percentile threshold is highly conservative in estimating a species tolerance for each predictor, which has been more commonly used (Liu et al. 2011). Half of the records were used for model calibration, and the other half were used for AUC and omission rate test. In the end, the overall occurrence was used to calibrate the model for exhibiting *P. japonensis*.

For *P. nagaensis*, the 6 occurrence records were inappropriate for a typical model evaluation approach involving partitioning the data into training and testing subsets. A modified jackknife approach specifically designed for small sample size was used (Pearson et al. 2007). In this method, independent Maxent models were generated iteratively, excluding one locality in each turn. The lowest suitability score of a presence point, or lowest presence threshold, for each model was then used to determine areas of predicted presence. The proportion of the training area predicted as present and the failure or success of the model to predict jackknifed points were then used to calculate the probability of the observed degree of coincidence between independent test data and predicted areas of suitability for *P. nagaensis* (Pearson et al. 2007). The overall occurrence was used to calibrate the model for exhibiting in the end.

### Niche identity and background test

Niche overlaps were measured by testing the similarities between habitat suitability predictions of the 2 species using ENM tools (Warren et al. 2008, 2010). Although only 6 occurrence records were available for *P. nagaensis*, which might lead to low significance, the 6 records were widely distributed and could represent the geographic range of the species’ distribution. The Schoener's *D* (Schoener 1968) and Warren's *I* statistic (Warren et al. 2008) were used because they were directly based on suitability scores and have been widely used for niche overlap measurements (McCormack et al. 2010; Hawlitschek et al. 2011; Peterson 2011). The metrics *D* and *I* were calculated by taking the difference between species in suitability score at each grid cell. The two metrics ranged from 0 (species have completely discordant ENM) to 1 (species have identical ENM) (Warren et al. 2010).

**Table 2.**
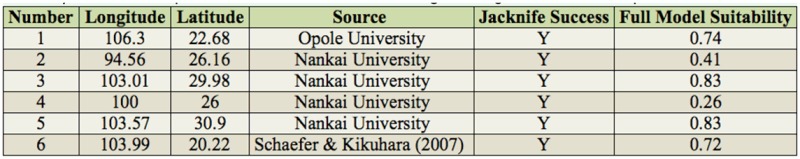
Six occurrence records for *Parastrachia nagaensis*, together with the model's success in predicting the excluded point in question, and the suitability score of each point in Maxent models trained using all *P. nagaensis* occurrence points.

Niche identity and background tests were then performed to determine whether the ENM generated for the 2 species were identical or exhibited significant difference, and whether these differences were caused by the environmental feature spaces. The niche identity test works by pooling actual occurrence points and randomizing their identities to produce 2 new samples with the same numbers of observations as empirical data. Niche overlap values generated by the actual occurrence data were then compared with those generated by the empirical data. The background test was used to ask whether the 2 species were more or less similar than expected based on the differences in the available environmental backgrounds. It works by comparing actual niche overlap with those generated using points drawn at random from the region defined as environmental background for one of the species (Warren et al. 2008, 2010). The minimum squares that covered all the occurrence of *P. japonensis* or *P. nagaensis* were set as the backgrounds. This disjunct disposal fit well the two species’ general dispersal patterns (Schaefer et al. 1988, 1991), and could represent their geographic distribution range. 500 replicates were used for both the identity and background test in ENM tools (Warren et al. 2010).

## Results

### Geographic distribution

**Figure 1.**
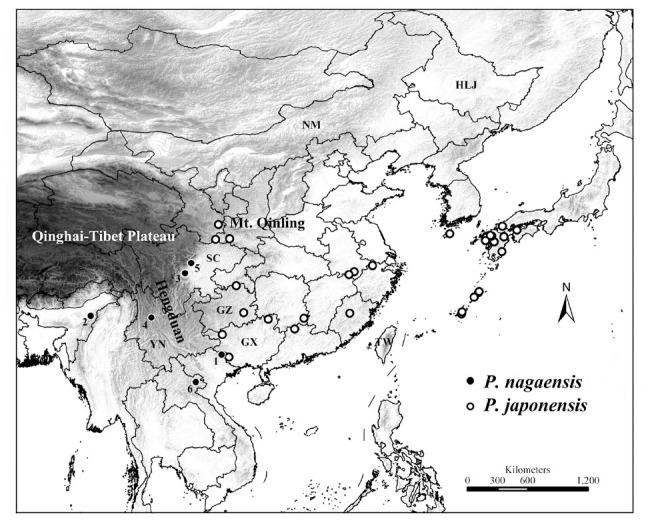
Geographic distribution of *Parastrachia*. Low to high altitudes are shown as a ramp from white to black. The Chinese province names in the text were simplified on the map (NM: Inner Mongolia, HLJ: Heilongjiang, SC: Sichuan, GZ: Guizhou, GX: Guangxi, YN: Yunnan). Numbers beside black dots indicate the *P. nagaensis* records (1 : Vietnam, New country record, 2: India, 3: Ya'an, China, 4: Yunnan, China, 5: Mt. Qingcheng, China, 6: Laos). High quality figures are available online.

In 2007, Schaefer and Kikuhara mapped the distribution of the genus, and reported a new country record of *P. nagaensis* in Laos, which extended the known species’ range about 300 km to south. Some records appeared on the map with unidentified specimens from China. Our results suggest that some of them were *P*. *nagaensis*, and the others were *P. japonensis* (Figure 1). *P. japonensis* showed a continuous distribution from Hengduan region to southern Japan with the north extended to western Mt. Qinling, while *P. nagaensis* exhibited a cryptic habitat with sporadic distributional records (Figure 1).

### Principle component analysis

**Figure 2.**
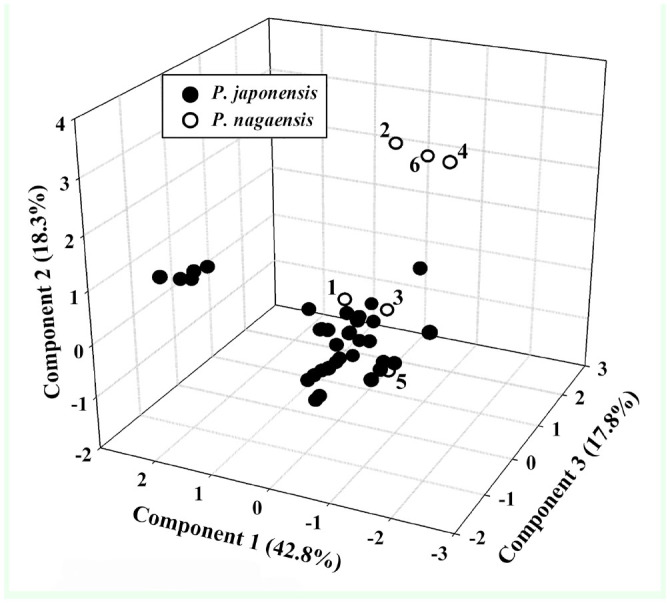
Principal component analysis of 13 variables associated with occurrences of *Parastrachia japonensis* and *P. nagaensis*. Symbols represent the insects’ occurrence in reduced 3 dimensions. High quality figures are available online.

PCA of pooled environmental variables revealed reduced significant components, defining a realized niche space occupied by *P*. *japonensis* and *P. nagaensis*. The first 3 components of the PCA were significant, and together explained 72.94% of the overall variance. The first component (PC-1) was related to temperature and precipitation, mainly contributed to by annual mean temperature, minimum temperature in the coldest month, annual precipitation, and precipitation in the driest month. The second component (PC-2) was associated with the lowest weekly radiation. The third component (PC-3) was less clearly associated with a single dimension (Table 1). Much niche space of *P. nagaensis* was differentiated from *P. japonensis* along the second and third components in the 3-dimensional plot, suggesting niche space might be diverged between the 2 species (Figure 2).

### Ecological niche modeling

**Figure 3.**
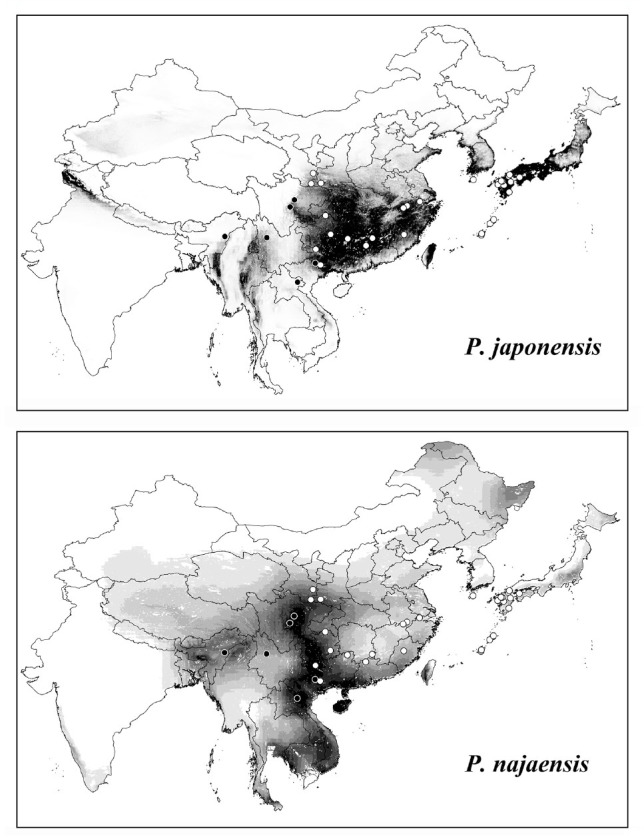
Potential distributions of *Parastrachia japonensis* and *P. nagaensis* based on Maxent. Model predictions from low to high suitability are shown as a ramp from white to black. White and black dots indicate the occurrence of *P. japonensis* and *P. nagaensis*. High quality figures are available online.

**Figure 4.**
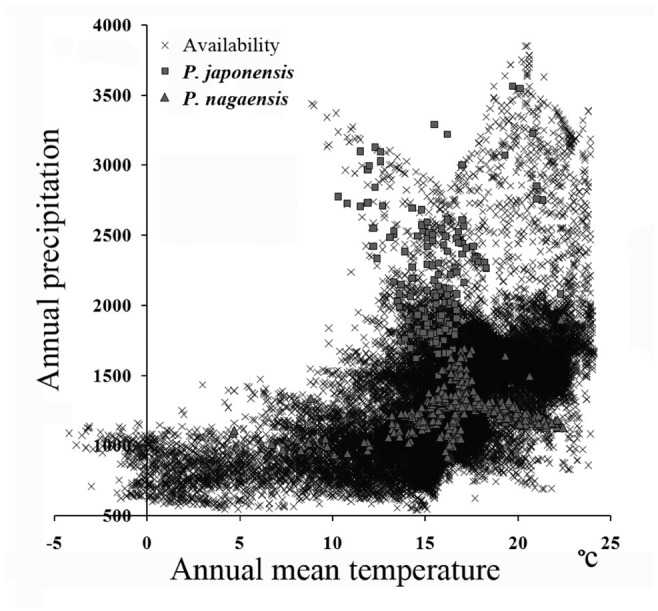
Two-dimensional visualization of the ecological niche of *Parastrachia japonensis* and *P. nagaensis*. Model predicted presence of *P. japonensis* (square) and *P. nagaensis* (triangle) according to the combinations of climate variables and the final predictions. High quality figures are available online.

The model output of *P. japonensis* showed good performance compared to random expectation (AUC = 0.82); the omission rate at the conservative threshold of the tenth percentile training presence was 33.3%. The model of *P. nagaensis* as measured by the Pearson jackknife-based test procedure was significantly better than random expectations (*p* > 0.01) (Table 2). The suitability score at each occurrence point for *P. nagaensis* ranged from 0.26 to 0.83, while suitability for *P. japonensis* ranged from 0.06 to 0.95. Areas in central southern China and southern Japan showed high suitability for *P. japonensis*, while the eastern Taiwan, mainland of South Korea, and northern Japan were also suitable (Figure 3). Highly suitable areas identified by the model of *P. nagaensis*, including the southeastern Qinghai-Tibet Plateau, western Mt. Qinling, Guizhou and Guangxi Provinces, eastern Indochina, and the disjunct areas in Heilongjiang and Iner-Monglia were also identified as suitable (Figure 3). Significant variables identified by the jackknife test for *P*. *japonensis* included precipitation in the driest month and the lowest weekly radiation. The jackknife test for *P. nagaensis* showed that highest weekly radiation was the most importantvariable. In the ecological dimensions, the 2 species occupied 2 distinct climate spaces (Figure 4), *P. nagaensis* occupied climate spaces of lower precipitation and higher temperatures compared to *P. japonensis*, suggesting it is more tolerant to dry and high temperature conditions.

### Niche identity and background test

**Figure 5.**
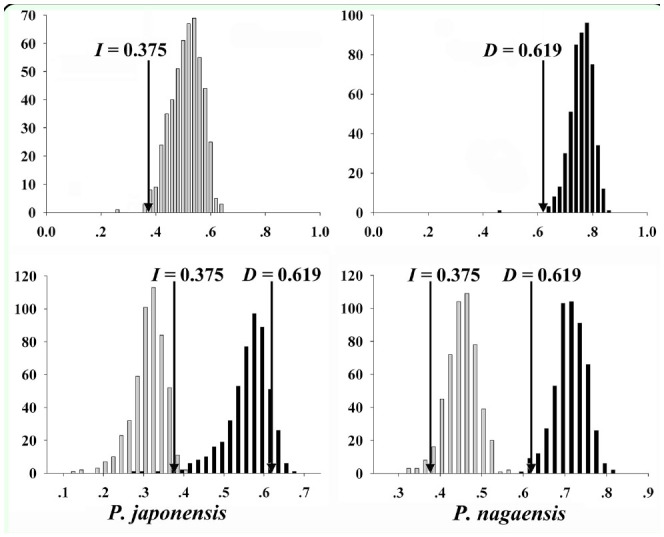
Results of niche identity (upper two panels) and background tests (lower two panels). Gray and black columns represent niche overlap values (X-axis) of D and *I* created in replicates of identity and background tests. Arrows indicate actual niche overlap distributed in the frequency (Y-axis) of the replicates. For the background tests, results are given both for *Parastrachia japonensis* (compared to the background of *P. nagaensis*) and *P. nagaensis* (compared to the background of *P. japonensis)*. High quality figures are available online.

The Schoener's *D* and Warren's *I* were 0.375 and 0.619 respectively for the actual niche overlap. Values of *D* were generally lower than those of *I*. In the identity test, the niche overlaps of *D* and *I* were 0.500 ± 0.056 and 0.750 ± 0.041 for the random resample occurrence data. The actual niche overlaps (D and I) were quite different from those of the random data (Figure 5), were outside the 95% confidence intervals, and thus were significant. In the background test, the focal species *P. nagaensis* with the background of *P. japonensis* showed that the *D* and *I* were 0.438 ± 0.037 and 0.708 ± 0.036, and the focal species *P. japonensis* with the background of *P*. *nagaensis* showed that the *D* and *I* were 0.297 ± 0.040 and 0.554 ± 0.055. Although actual niche overlap values appeared at the lower end of the distribution of overlaps from randomly drawn points (Figure 5), the actual niche overlap (*D* and *I*) was inside the 99% confidence intervals of the background test results and therefore were not significant.

## Discussion

### Model interpretation

Limitations on the material and methodology employed in this study need to be addressed here. Due to cryptic habits, locally restricted distributions, or low sampling effort, the occurrence data of *P. nagaensis* was limited to only 6 available records. Ecological dimensions could not be directly compared and statically tested using scarce occurrence data, and model extrapolation based on such data should be performed with caution (Stockwell and Peterson 2002; Pearson et al. 2007; Wisz et al. 2008; Peterson 2011). In our study, the model output of *P. nagaensis* could not be interpreted as predicting actual limits of the species’ range, but could identify regions that had similar environmental conditions to areas where the species was known to occur (Pearson et al. 2007), while the model output of *P*. *japonensis* could be interpreted as the suitability index in the geographic space.

### Geographic distribution

Southwestern and southern China is one of the most species-rich regions in the world (Dai et al. 2011). Potential distributional areas inferred by ENM for the genus *Parastrachia* might be useful for future field surveys in these areas. ENM seeks to characterize the realized niche in a way that approaches the fundamental niche without consideration of the species’ dispersal ability or biotic interactions (Soberón and Peterson 2005). In some areas identified as suitable, like eastern Taiwan, the mainland of South Korea, and northern Japan for *P. japonensis*, or the Heilongjiang and Iner-Monglia for *P. nagaensis*, the species were absent, potentially due to the dispersal limits or lack of availability of suitable host plants. The distribution of *P*. *nagaensis* also occurred in the indo-Burma and South Central China hotspot (Mayers et al. 2000). Species in hotspots tend to be scarce within their range, which increases their probability of extinction (Brown 1984; Gaston 1994). The potential distribution suggests this insect's range could extend beyond its presently known distribution to further south. Further investigation could aid in forming a more complete picture of the genus‘ distribution, which in turn could aid in their conservation, particularly for *P. nagaensis*.

Schaefer et al. (1988, 1991) suggested that *P*. *japonensis* might be the more plesiomorphic of the 2 species, and it might more closely resemble the 2 species‘ ancestor. They hypothesized that *P. nagaensis* was the western descendant of a more widely ranging ancestor, while *P. japonensis* was the more eastern descendant. The mapped distribution supported the above hypothesis and further filled in the blank region between southwestern China and southern Japan (Figure 1). The current distribution of *P. japonensis* might be the result of population expansion from the Hengduan region, as the Hengduan region has acted as a refuge for hosting many ancient species (Huang et al. 2006). Recent studies showed close a relationship of the genus *Parastrachia* with the Ethiopian genus *Dismegistus* Amyot and Serville (Grazia et al. 2008; Pluot-Sigwalt and Lis 2008), however, the genus *Dismegistus* was poorly studied, and the divergence of these 2 lineages might be related to historical geographic events.

### Ecological niche

Significant variables identified by the PCA and ENM were in accordance with the general biology of *P. japonensis*. The habitat of *P*. *japonensis* was restricted to early-stage secondary forests in foothill areas (Filippi et al. 2001). This insect is a specialist feeder that utilizes drupes of the Olacaceous tree, *Schoepfia* *jasminodora*, as its sole food resource (Tachikawa and Schaefer 1985). It requires good shade and usually forms aggregations on the underside of hard waxy leaves for summer estivation (Filippi et al. 2001). In winter, the consolidated, massive aggregations enter into the large soil holes under leaf litter for hibernation (Hironaka et al. 2005). In the PCA, the first component was associated with temperature and precipitation, whereas the second was related to sunshine. In the ENM, 2 significant variables were identified for *P. japonensis*, including precipitation in the driest month and the lowest weekly radiation. The jackknife test for *P. nagaensis* may not be reliable due to its small sample size. The host plant and habitat for *P. nagaensis* are currently unknown. *P*. *nagaensis* is usually distributed in mountain areas of high altitude (500–3500 m a.s.l.). Schaefer et al. (1991) outlined the general distribution of Olacaceous trees and *Schoepfia* spp. in China, Japan, and India, and suggested that *S. jasminodora* may be the host of *P. japonensis* in China, and some other *Schoepfia* species may be the host(s) of *P. nagaensis*. Further field investigations may reveal the real host plant and cryptic habits of *P. nagaensis*.

### Niche divergence

Reduced niche dimensions identified by the PCA occupied by the 2 species allows investigation of niche conservatism and differentiation. In 3 dimensions, *P. nagaensis* showed a shifting niche space from *P. japonensis* along the second and third components, and much of the niche space of *P. nagaensis* was differentiated (Figure 2). The second component was associated with radiation while the third was less clearly associated with a single dimension (Table 1). The visualization of modeled niche space in ecological dimensions also suggests the 2 species occupied 2 climate spaces, with *P. nagaensis* more tolerant of dry and high temperature condition (Figure 4). In the identity and background test, a significant niche identity test indicates significant differences in niches between the 2 species; however, the possibility that this differentiation is primarily driven by allopatry cannot be ruled out. Although the 2 species do not occupy identical habitats, the background test suggests the differences between their ENMs could simply be due to the differential availability of habitat in the different regions that they occupy.

Soberón and Peterson (2011) proposed to adopt the fundamental niche, potential niche, and realized niche (or existing fundamental niche) to elaborate the ENM based niche differentiation issue. They insist that the ENM characterizes somewhere between the realized and potential niche. The above observed niche difference can be considered as the realized niche difference, but may not indicate that the 2 species are actually biologically different at all (i.e., they may not have any significant differences in their fundamental niche), since the fundamental niche is generally and not fully manifested in a certain environment (Soberón and Peterson 2011). The observed niche difference might also be due to high dimensionality of the environmental variables and the limited distributional records (Peterson 2011). Although we tried to reduce such negative effects in niche modeling, the niche divergence analysis here is tentative, based on the limited records available for *P. nagaensis*. The results suggest that the 2 species are occupying different habitats, but that may be driven primarily by availability (Warren et al. 2008).

ENM offers useful information for the identification of poorly known species. Evidence of niche divergence had been explored for species delimitation (Raxworthy et al. 2007; Rissler and Apodaca 2007; Leaché et al. 2009; Martínez-Gordillo et al. 2010), and many studies have shown its coincidence with DNA sequence data (Fitzpatrick et al. 2007; Waltari et al. 2007; Flanders et al. 2010; Hawlitschek et al. 2011). Somatic characters for discriminating between the 2 species sometimes appear unstable (Schaefer and Kikuhara 2007); however, genital characters support their distinct existence (Schaefer et al. 1988, 1991; Schaefer and Kikuhara 2007). ENM for species delimitation is quite useful for taxonomic groups with low vagility, localized endemism, and poorly known distribution (Raxworthy et al. 2007), which might be useful for the genus *Parastrachia*.

### Conclusion

Parastrachiidae is a small stinkbug family containing one genus and two species, *P. japonensis* and *P. nagaensis*. The former showed a continuous distribution from the Hengduan region to southern Japan, with the north extended to western Mt. Qinling, whereas the latter exhibited a cryptic habitat with sporadic distributional records. The potential distributions of the 2 species were anticipated using ENM, which may prove useful in searches for new populations and in conservation of this genus. The ecological dimensions occupied by the 2 species were identified and compared, and the comparison suggested the 2 species do not occupy identical habitats. But, the differences could simply be due to the differential availability of habitat in the different regions they occupy.

## Abbreviations:

AUC,: area under the curve;
**ENM,**: ecological niche modeling,
**PCA,**: principal component analysis
